# Detection in influx sources and estimation of microplastics abundance in surface waters of Rawal Lake, Pakistan

**DOI:** 10.1016/j.heliyon.2022.e09166

**Published:** 2022-03-24

**Authors:** Atif Bashir, Imran Hashmi

**Affiliations:** Institute of Environmental Sciences and Engineering, School of Civil and Environmental Engineering, National University of Sciences and Technology, Islamabad, 44000, Pakistan

**Keywords:** Microplastics, Rawal Lake, Plastic pollution, Freshwater, Influx sources, Surface waters

## Abstract

The ever-growing production, usage and poor waste management practices of plastics are causing microplastics intrusion in freshwater environments all over the world. The identification of inflow processes and sources is equally important as the assessment of microplastic concentrations in freshwater. This study reports microplastic presence in the influx sources and provides an overall estimation of microplastic concentration in the surface water of a freshwater reservoir, Rawal Lake, Islamabad. In the current study, six major tributaries of Rawal lake were assessed for microplastic presence, out of which four tributaries showed microplastic contamination. Microplastics concentration in the lake ranged from 6.4 ± 0.5 particles/m³ to 8.8 ± 0.5 particles/m³. All the identified microplastics in tributaries and lake were secondary except granules. The prominent shape of microplastics among the studied waters was film, with transparent being the most frequent plastic-type according to color. Polyethylene (LDPE and HDPE) were the dominant type of microplastics found in the lake and the tributaries. More than 72% of microplastics had a size of 0.3–0.1 mm. This study provides a better understanding of the extent of microplastic pollution assessment in a freshwater lake with equal emphasis on microplastic presence in influx sources and the relationship of microplastics with fundamental water quality indicators (pH, temperature, dissolved oxygen, and biological oxygen demand), which may be beneficial in impeding the introduction of microplastics at sources.

## Highlights

• Microplastics were estimated in an artificial freshwater reservoir.
• Microplastics were present in influx sources in regions nearer to main city.
• Finer sized microplastics were abundant.
• Polyethylene was the predominant microplastic in the Lake and tributaries.

## 1 Introduction

Plastics, being one of the most commonly used synthetic products in everyday human life, are becoming an omnipresent pollutant in every environmental matrix, especially in the aquatic environment (Khan et al., 2018; Barboza and Gimenez, 2015). The overall production of plastics has soared to 348 million tons (PlasticsEurope & PEMRG, 2018). Plastics, once entered into the environment, tend to persist for hundreds of years under natural conditions because of their stable structures (Hermabessiere et al., 2017; Li et al., 2016)**.** However, under the effect of UV radiation, weathering processes, and environmental aging, plastics are disintegrated into smaller fractions, called microplastics. Additionally, these smaller size fractions are also produced by industries like cosmetics. The former type is termed "secondary," and the latter is considered "primary microplastics." (Browne et al., 2011; Farrell and Nelson, 2013; Sundt et al., 2014). These microplastics particles may be found approximately in all aquatic ecosystems as well as in drinking water (Browne et al., 2011). Since the start of the century, the point of focus in microplastic studies has been on the marine environment (Thompson et al., 2004; Cózar et al., 2014; Tanaka et al., 2015; Song et al., 2015; Pedrotti et al., 2016; Suaria et al., 2016; Cincinelli et al., 2017; Fok et al., 2017; Zhang et al., 2017). But in recent years, the importance of microplastics in the freshwater environment has also attained prominence (Castañeda et al., 2014; Wagner et al., 2014; Baldwin et al., 2016; Su et al., 2016; Langen et al., 2017; Jiang et al., 2019; Kataoka et al., 2019). Although several studies have been reported, this field of study still lacks a standardize sampling and processing methodology that hampers the effective comparison of results on a global scale (Bordós et al., 2019). Multiple research groups across the globe use multiple approaches for microplastic studies (Prata et al., 2019).

According to the findings, the majority (more than 95%) of plastic litter persist in the terrestrial environment. Hence, microplastic pollution of inland waters is more fundamental than marine waters as freshwater systems act as a transport network for oceans, transporting 70–80% of marine debris (Bowmer and Kershaw, 2010; Andrady, 2011; Jambeck et al., 2015). High concentrations of microplastics have been reported in many lakes and rivers (Table SD2). In many freshwater systems, microplastic pollution are of similar magnitude or even more abundant than some oceanic environments (Zhang et al., 2018). The reported abundance of microplastics in freshwater systems ranges from 0.00031 particles/m^3^ to 10200 particles/m^3^ (Li et al., 2018).

Land environments are the leading source of marine plastic debris. According to estimates, 5.25 trillion macro and micro plastic particles have been dumped into the ocean (Alfaro-Núñez et al., 2021). The investigation of the characteristics and locations of microplastic pollution, as well as the inflow processes of freshwater systems, proves to be an effective method for carrying out a source analysis of oceanic microplastic pollution and developing subsequent local policies (Choi and Lee, 2018; Iacovidou et al., 2019). “Cutting off” at the freshwater sources is the best way to diminish the microplastics contamination in oceans and mitigate subsequent risks. The key sources of microplastics intrusion in freshwater include microbeads from cosmetics, waste from plastic industries, broken down litter, and scuffing from tires and textile products (Barnes et al., 2009; Boucher and Friot, 2017; Lechner et al., 2014; Lechner and Ramler, 2015; Scudo, 2017; Sommer et al., 2018). A few studies have been conducted to examine the influence of atmospheric traits and physicochemical parameters on microplastics concentrations (Barrows et al., 2018; Hitchcock and Mitrovic, 2019; Kataoka et al., 2019; Luo et al., 2019). Domestic wastewater, littering, surface run-off, landfill leachate, and atmospheric deposition are the other key pathways of microplastic entrance into freshwater (Dris et al., 2016; Gandara e Silva et al., 2016; He et al., 2019; Murphy et al., 2016; Nizzetto et al., 2016; Peters and Bratton, 2016; Wei et al., 2019).

The present study exercises a similar approach of highlighting the importance of “cutting off at the source” by providing a better understanding of microplastic input in a freshwater system, Rawal Lake in Islamabad, through the detection of insertion points of microplastics in the influx sources. The current study aims to better understand the effects of land use along tributaries and a lake on microplastic contamination in a country where plastic accounts for 65% of total waste generated (WWF Pakistan, 2020). A similar study at Rawal Lake (Tahira et al., 2020) provides initial evidence and baseline data for microplastics in Pakistan but lacks a consistent and standard sampling approach. This present study attempted to provide an overall estimation of microplastic concentration in surface water of the lake, their distribution and characterization, identification of insertion points of microplastics in tributaries, establishment of a link between microplastics present in the lake and in tributaries, and the nexus of microplastics’ presence with fundamental water quality indicators (pH, temperature, dissolved oxygen, and biological oxygen demand), along with the idea of facilitating the establishment of a standardized sampling approach at least at the local level to develop coherence between results. The results of our study may be utilized as a useful reference for future research in Pakistan and may expand the current knowledge of microplastic pollution.

## 2 Materials and methods

### 2.1 Study area and sampling method

**Figure 1:**
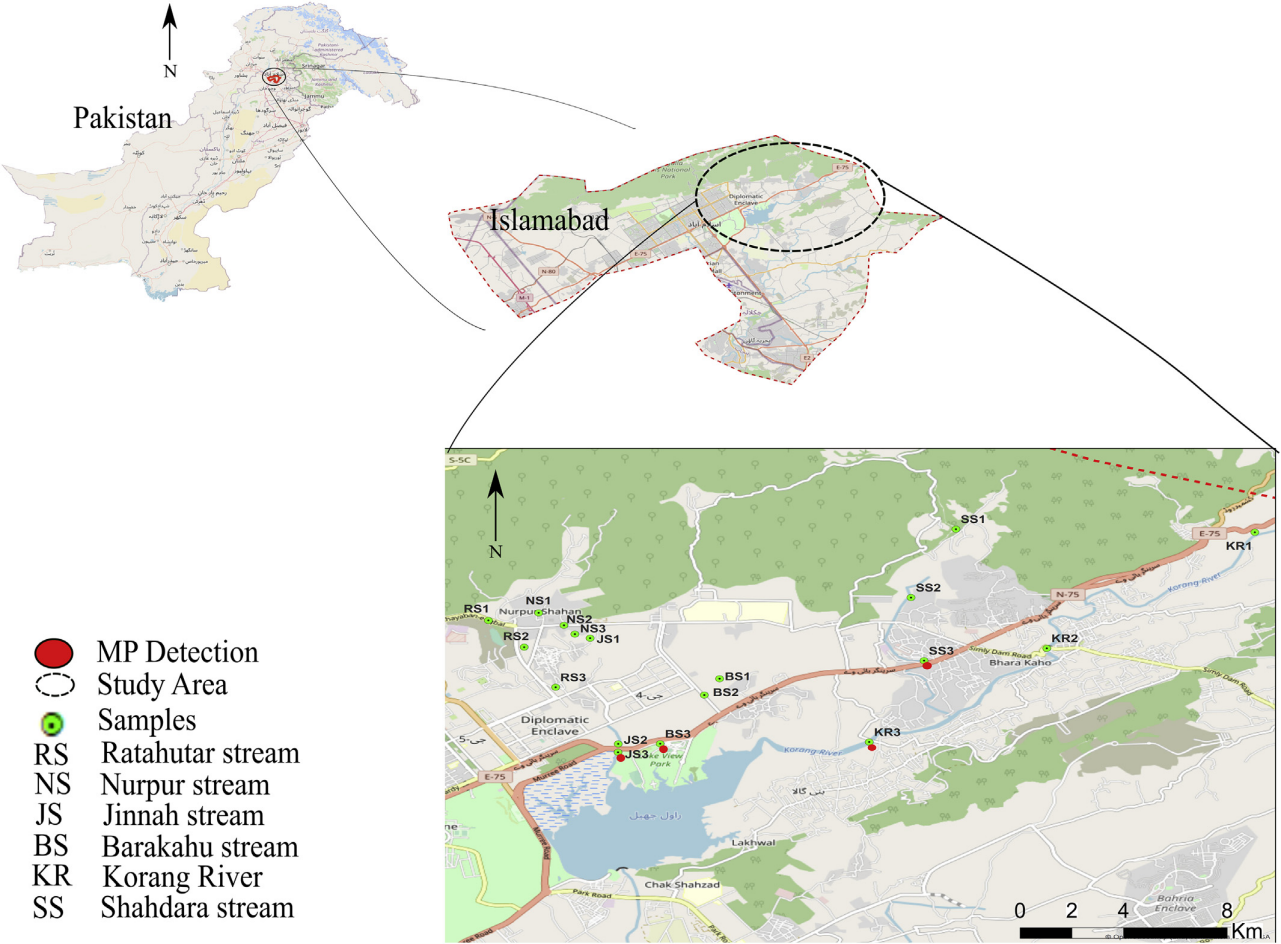
Geographic locations of sampling points and, Microplastics detection in Rawal Lake tributaries.

Rawal Lake lies (33°42′09.6″N 73°07′34.0″E) in Islamabad (Figure 1). The lake acts as an artificial reservoir and a major source of drinking water for Islamabad and Rawalpindi by providing 83,279 m^3^ of water per day (Saeed and Hashmi, 2014). The total surface area of the lake is about 8.8 km^2^ with a max depth of 31 m. The lake receives inflow from 47 major and minor tributaries and has a catchment area of 268 km^2^. Human settlements along the tributaries and lake, waste dumping, recreational activities, waste from poultry farms, and agricultural activities are identified as prospective sources of pollution (Pakistan Environmental Protection Agency, 2004).

After an extensive walkthrough survey, six major tributaries contributing to the inflow of Rawal Lake were selected based on accessibility and their major contribution in the inflow, i.e., Shahdara stream (SS), Barakahu stream (BS), Nurpur stream (NS), Ratahutar stream (RS), Jinnah stream (JS), Korang River (KR). 18 sampling points (upstream, midstream, downstream points at each stream) were identified at the six selected tributaries by the global positioning system to gain a better understanding of the insertion points of microplastics in inflowing water (Table.1). Three replicates (one from each side and one from the middle of tributary) were collected at each sampling point and pooled as a single sample. A grab sampling approach was used by dipping the 1 L sampling bottle in surface water and slightly tilting it upright to completely fill the bottle with the surface water (Barrows et al., 2017; Boyle and Örmeci, 2020; Sartain and Sparks, 2020).

**Table 1:** Geographic Locations of the sampling sites.

Sampling Locations	Sampling Points	No. of Samples^a^	Coordinates (Latitude, Longitude)^b^	
Ratahutar stream	Upstream (RS1)	3	33.745394	73.100868
	Midstream (RS2)	3	33.739863	73.106787
	Downstream (RS3)	3	33.731529	73.111961
Nurpur stream	Upstream (NS1)	3	33.746944	73.109166
	Midstream (NS2)	3	33.744362	73.113333
	Downstream (NS3)	3	33.742555	73.115124
Jinnah stream	Upstream (JS1)	3	33.741682	73.117657
	Midstream (JS2)	3	33.719763	73.122278
	Downstream (JS3)	3	33.718022	73.122256
Barakahu stream	Upstream (BS1)	3	33.733300	73.139004
	Midstream (BS2)	3	33.729909	73.136472
	Downstream (BS3)	3	33.719790	73.129209
Shahdara stream	Upstream (SS1)	3	33.76436	73.17797
	Midstream (SS2)	3	33.75014	73.17055
	Downstream (SS3)	3	33.73706	73.17272
Korang River	Upstream (KR1)	3	33.763689	73.227229
	Midstream (KR2)	3	33.739620	73.192946
	Downstream (KR3)	3	33.720141	73.163679

a Triplicates collected at each sampling point, deemed as a single sample.

b Coordinates recorded through GPS.

The four selected water quality parameters (pH, temperature, dissolved oxygen, and biological oxygen demand) were assessed for water samples from the tributaries by using Standard Methods (APHA, 2017) for sample collection and analysis.

For sampling at the lake, a build-it-yourself research trawl, the LADI (Low-tech Aquatic Debris Instrument) trawl was used, which is an effective substitution for the standard Manta trawl used in microplastic studies and reportedly gives similar efficiency (Coyle et al., 2016) Figures SD1 and SD2. The trawl was built with 100 μm mesh to allow for a more accurate estimation of microplastics since smaller microplastics tends to escape the standard 333 μm mesh (Wang et al., 2017; Prata et al., 2019). Sampling was conducted during calm atmospheric conditions.

**Figure 2:**
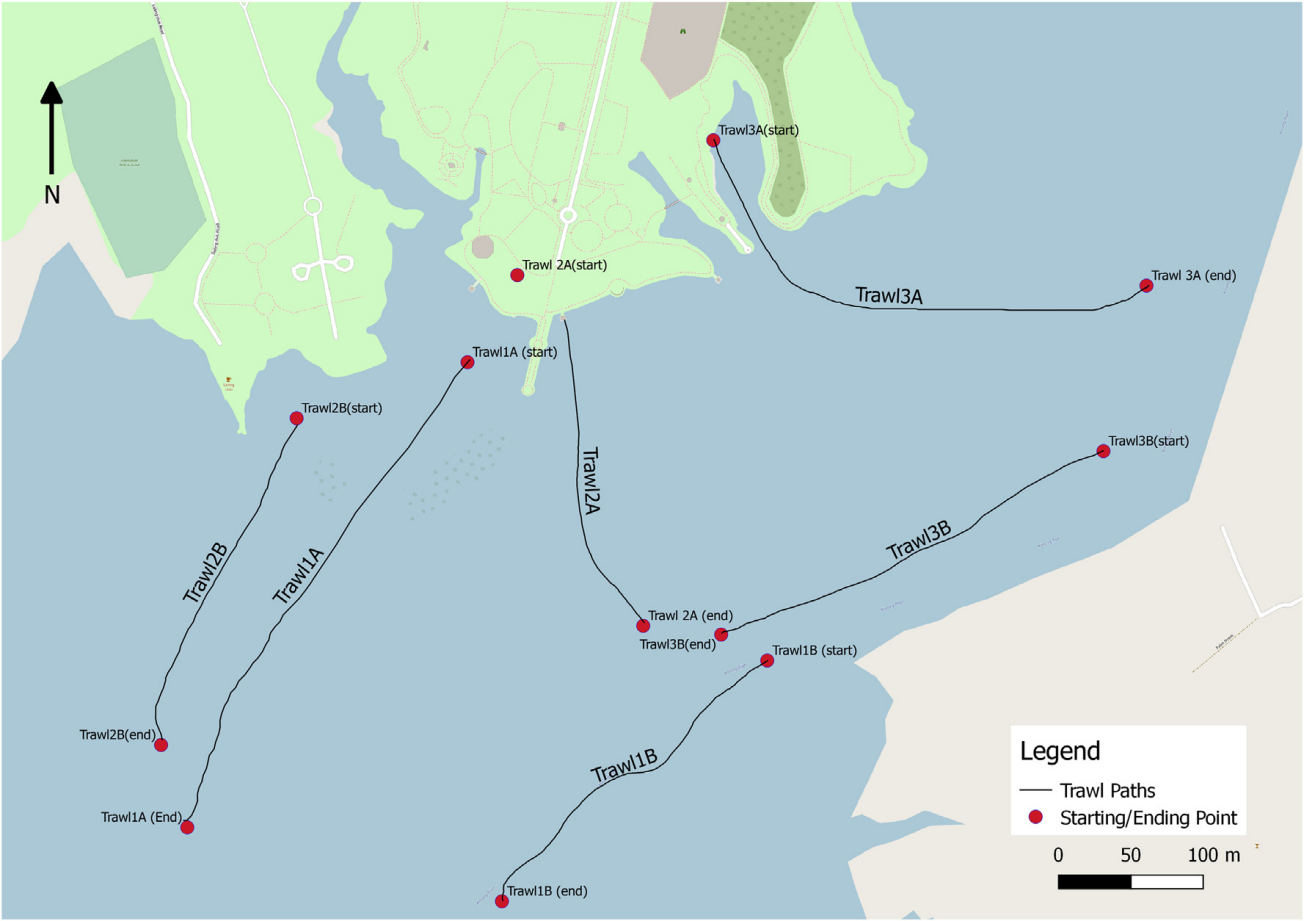
Towing points of LADI trawl in Rawal Lake (Two hauls during each sampling visit).

Towing points were selected hypothetically with the idea to exhibit true representativeness, few portions of the lake nearer to the dam were out of bound for boating. Towing time varied between 7–13 min (UNEP/MAP, 2019), and tow speed ranged between 1.6–2 ms^−1^. The trawl was kept on the side of the boat to avoid the turbulence caused by the boat, and the mesh was closely monitored for clogging during the towing. If any clogging was observed, towing was stopped, and cod-end was retrieved and rinsed, and the sample was transferred to a cleaned glass beaker and covered with aluminum foil. The total distance covered by the trawl (see ​Table 2) was estimated by recording the coordinates of starting and ending points through GPS (Figure 2). Volume of water was estimated by using the formula of volume flow rate Eq. (1), and the total time of a tow.

(1) Q = vA

**Table 2:** Microplastics sampling visits at Rawal Lake.

Sampling Visits (n = 2)^a^	Sampling dates	Sampling hauls	Distance (m)
Trawl 1	10th Feb 2020	1A	750
		1B	560
Trawl 2	12th Feb 2020	2A	330
		2B	490
Trawl 3	14th Feb 2020	3A	630
		3B	590

a Two hauls during each visit.

v: velocity  A: cross-section area of trawl mouth

### 2.2 Sample processing and laboratory analysis

The analysis of the water samples in the laboratory was performed using NOAA laboratory methods for the analysis of microplastics (Masura et al., 2015) with some modifications. The sample processing consisted of wet sieving through stacked sieves of assorted sizes 5000, 300, 106 and 45 μm followed by wet peroxide oxidation with the addition of pure sodium chloride and density separation. After sieving, plastic, and organic fraction > 5 mm were carefully rinsed under 4× magnifying glass and discarded. The remaining fractions <5 mm were rinsed, removed, and subjected to wet peroxide oxidation with 30% H_2_O_2_ and 0.05 M Fe(II) as a catalyst at 75 °C until boiled, with the addition of 6 g pure NaCl per 20 mL of solution to exterminate the organic matter. After digestion, the reaction was allowed to cool down and subjected to density separation for 24 h which allowed microplastics to float while settling down the digested organics, which were drained later, and plastics were collected through a 45 μm customized sieve (Masura et al., 2015) instead of traditionally used filter papers in several MP studies, it makes the collection of MPs for further analysis easier, cost-effective, and reduces the chances of possible cellulose easter contamination and risk of perceiving microfibers of filter paper as microplastics from the use of filter papers. After, filtration the sieve was oven-dried at 50 C for 24 h to eliminate all the moisture. Once dried, the sieve was examined under a stereomicroscope **(**Levenhuk 3ST) for visual inspection. Any possible remains of organic material were removed and microplastic particles were transferred for further observation and pictorial substantiation to an Optical microscope (Carl Zeiss Axio-Lab A1) and HD-color camera (2560 × 1920-pixel) with the operating system (IDS-UI-1480LEC-HQ USB2.0).

The numbers, color, shape, and size of each microplastic fraction were recorded. The concentration was expressed as the number of particles/m^3^ (UNEP/MAP, 2019). Microplastics were categorized according to the meticulous criteria stated in literature (Hidalgo-Ruz et al., 2012; Nor and Obbard, 2014) and principally based on their morphological properties (shape and color). According to shape, microplastics were categorized into film, fiber, granule, foam, and pellet as cited in the literature (Free et al., 2014; Mani et al., 2015; Zobkov and Esiukova, 2017; UNEP/MAP, 2019).

A Fourier transform infrared spectrometer PerkinElmer-L1280133-Spectrum 100 Model, in transmission mode 350 cm^−1^–4000 cm^−1^ was utilized for identifying the polymer composition by prudently selecting particles of different shapes and structures from all the microplastic fractions retrieved from the sample processing stages. The number of scans was set to 1 and the resolution was set to 4 cm^−1^. 3% of total extracted microplastics from each sampling visit was subjected to FTIR examination. The spectra were compared with multiple online databases and IR tables (John Wiley and Sons, 2020; Thomas, 2021) for composition validation. It should be noted that retrieved microplastics were contaminated with organic or chemical components or coloration of plastic products may cause distortion in the IR spectra of the sample (Horton et al., 2016) as compared to the pure compound spectra in the library and databases. Therefore, current study did not set any specific coinciding limit with the reference spectra and inspected the spectra for peaks as stated by (Kataoka et al., 2019). The graphical overview of the adopted methodology is presented in Figure SD3.

### 2.3 Quality assurance and quality control

All the preventive measures in the course of MP studies in the literature (Lusher et al., 2014; Rocha-Santos and Duarte, 2015; Wang et al., 2017; Rodrigues et al., 2018) were considered in the current study. During the sample collection and processing, the use of any plastic material was avoided. All the glass beakers/bottles were rinsed thrice with the distilled water prior to usage. All the materials were immediately covered with aluminum foil after usage at each step. The cleaning of the workplace during each procedure was ensured by using natural sponge. Nitrile gloves, cotton lab coats were strictly used to avoid any potential interference with the sample collection and analysis. Microscopic examinations were set in secluded rooms with restricted air flow.

To inspect any conceivable contamination during the sampling and analysis, field blank test and laboratory blank test were carried out for distilled water, chemicals and equipment being used in sample collection and processing, and laboratory air (through vacuum filtration by sucking laboratory air for 1 h through a 0.45 μm glass microfiber filter paper) (Chae et al., 2015; Dubaish and Liebezeit, 2013; Nuelle et al., 2014; Wang et al., 2017). Each blank test was set in triplicate. No contamination of plastic particles was observed during blank testing. Hence, the entire process was designed to be as contamination free as possible with best available resources. 4 persons participated in the microscopic examination and characterization of microplastics to avoid any kind of biases and inaccuracy in reporting the results. As part of contamination control protocol, particles from nylon mesh in the sampling trawl were used to compare and monitor the number of plastic particles coming from the mesh as contamination and excluding them from the final counts. No nylon particle was detected throughout the study.

## 3 Results and discussion

### 3.1 Abundance of microplastics

Sampling at tributaries was conducted during November–December 2019. Out of 6 major tributaries selected, 4 (Jinnah, Barakahu, Shahdara and Korang) showed the presence of microplastics. Nurpur stream was apparently most polluted stream of all, but no microplastics were detected. which may be attributed to high sewage waste, algal growth, and resultant biofouling. In contrast to Nurpur stream, Ratahutar stream showed less pollution may be due to fewer human settlements nearby, and no microplastics were detected, possibly due to shallow water that did not allow microplastics to float and less plastic waste dumping. All of the 4 tributaries exhibited the presence in the downstream points (JS3, BS3, SS3, KR3) (Figure 1), with the concentrations ranging from 0.7–2.3 particles/Liter, which could be attributed to the increasing population downstream (Eriksen et al., 2013; Rodrigues et al., 2018; Wang et al., 2017) and less turbulence of flowing water, which allowed for the floating of plastics on the surface to be sampled. At the upstream points, water seemed clear with few signs of dumping and rare population settlements that were not connected to a proper sewer system in the vicinity, except for the Shahdara tributary, which has a recreational point located in the upstream region. However, waste dumping and human settlements became significant in midstream region and kept mounting as moved to the downstream area along with intrusion of animal feces in some of tributaries. Polyethylene (PE) and polypropylene (PP) were the only plastic types detected in the tributaries. Contrary to assumption, all the identified microplastics from the tributaries were of secondary origin.

**Figure 3:**
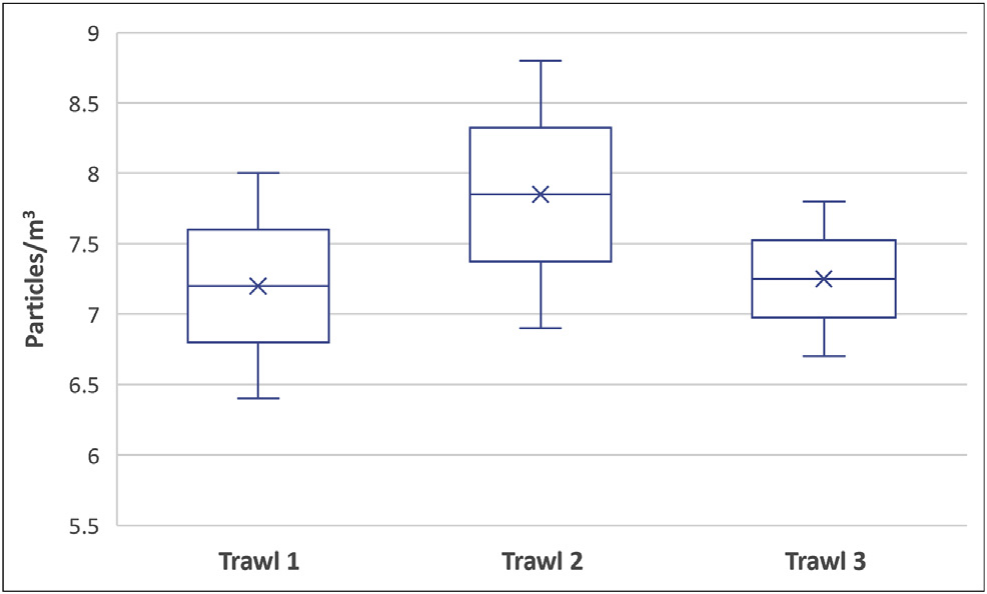
Microplastics (MP) concentrations in Rawal Lake.

Sampling at lake was conducted during the second week of February 2020. The surface water of lake indicated microplastics presence upon analysis. The concentrations ranged from 6.4 ± 0.5 to 8.8 ± 0.5 particles/m³ (Figure 3), which is considerably lower than the previous study at Rawal Lake and compared with many other lakes across the globe which were examined for microplastics (Bordós et al., 2019; Faure et al., 2015; Fischer et al., 2016; Gopinath et al., 2020; Tahira et al., 2020; Mani et al., 2015) (Table SD2).

After employing the most common sampling method with a finer mesh size and sampling a large volume (332 m^3^) of surface water, the results contradicted the assumption. This may be imputed to several factors, such as continuous everyday discharge of a large volume of water to twin cities, which did not allow microplastics to get concentrated within the lake and biofouling, which is highest during the summer months, along with adsorption of physical substances which may result in the submerging of microplastics in lake sediments (Corcoran et al., 2015; Frias et al., 2016; McCormick et al., 2014; Chen et al., 2019; Amaral-Zettler et al., 2021; Miao et al., 2021). Photo-oxidation, which is considered the most effective abiotic degradation phenomenon for polymers in freshwater systems (McKeen, 2013), degrades particles into nano-sized fractions and increases the polymer surface area, further assists in biotic degradation, adsorption of pollutants and increases the risk of bioaccumulation and ingestion in organisms (Acosta-Coley and Olivero-Verbel, 2015; Da Costa et al., 2016; Endo et al., 2005; Veerasingam et al., 2016). These nano-sized fractions may also easily escape through the micro-sized sampling mesh and may result in the underestimation of microplastics (Boyle and Örmeci, 2020). The initial evidence of microplastic presence in Rawal lake reported by (Tahira et al., 2020), which showed relatively much higher concentrations of 0.142 particles/0.1 L, also indicates that finer size microplastics are more abundant in Rawal lake, as samples were collected by beaker and filtered through Whatman GF/F (0.7 um × 47 mm). Extensive boating activity in the Rawal lake can also play its part in concentrating microplastics from surface water to the shoreline along with other environmental aspects (Wang et al., 2017; Rodrigues et al., 2018). For instance, trawls 1 and 3 points as shown in (Figure 2) are the areas where maximum of boating activity usually come about, and these areas showed relatively less microplastic concentrations than trawl 2 (Figure 3). The effect of boating may also be perceived in study by (Tahira et al., 2020), in which the majority of the sampling was done at lake shores, where microplastics may become concentrated, thus resulting in higher reported concentrations, and shore sediment analyses, also revealed higher microplastic concentrations than lake surface water.

### 3.2 Relationship of microplastics with water quality

Water quality indicators were monitored in the tributaries to observe the effect of pollution on microplastics concentration. The results of water quality parameters are given in (Table SD3). The presence of microplastics in the downstream region of most tributaries was in significant relation with dissolved oxygen and biological oxygen demand. Both of these indicators are used to show the pollution level of water. The results showed that the level of dissolved oxygen decreased, and biological oxygen demand increased as progressed towards the more polluted downstream regions, and these were the only points in tributaries where microplastics were detected (Figure 1). Similar results were reported by (Kataoka et al., 2019). However, pollution levels were apparently higher in Nurpur stream (Figure SD6) but no microplastics detection was observed, indicating the probability that presence of microplastics may not always associate with pollution levels.

### 3.3 Morphological properties of microplastics

**Figure 4:**
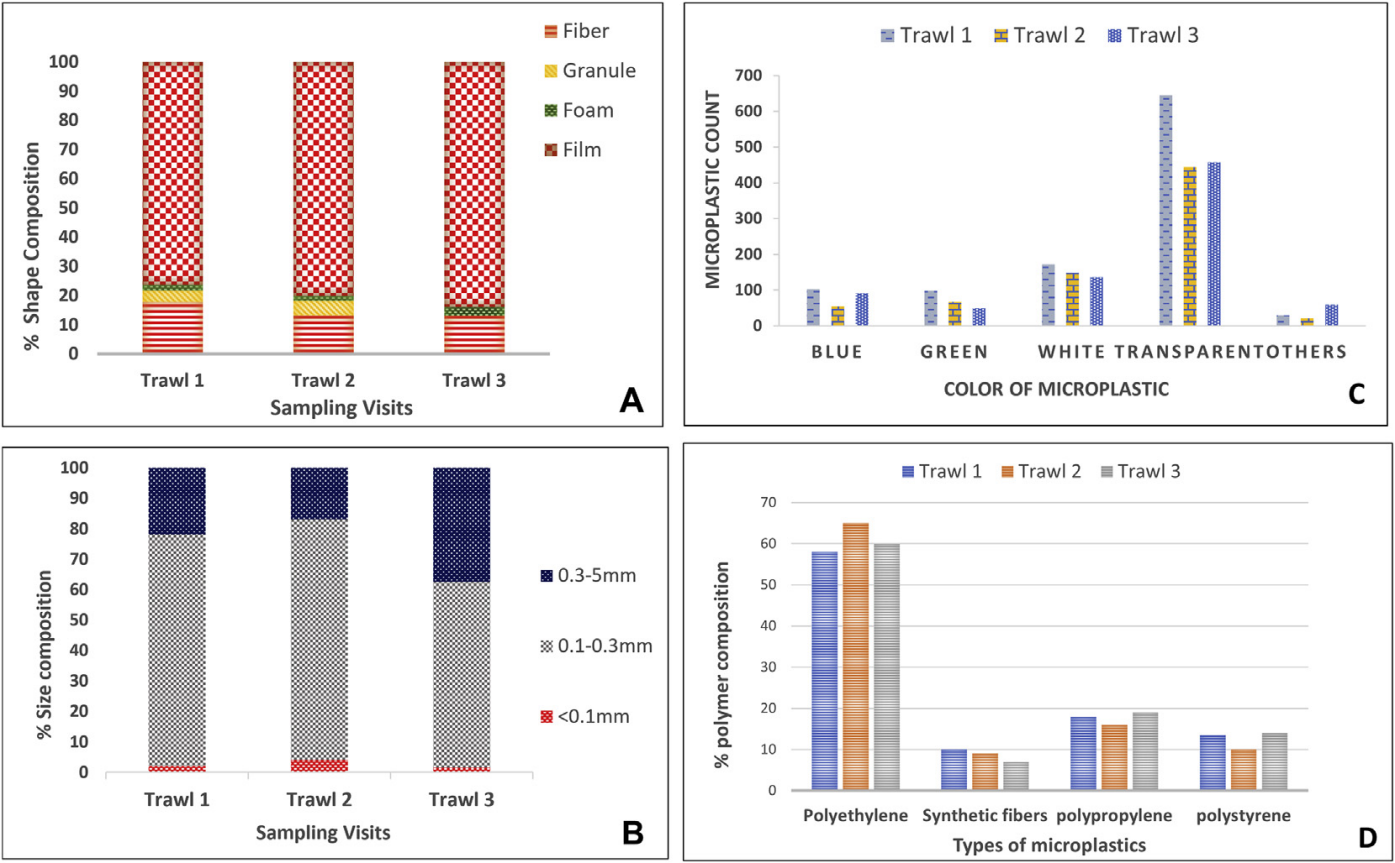
Composition and distribution of microplastics in (A)shape, (B) size (C) color and (D) polymer type.

Altogether, detected microplastics were in the size range of 0.045 < 5 mm as reported in previous lakes studies (Eriksen et al., 2013; Free et al., 2014; Tahira et al., 2020) with 74.5% lying below the range of 0.33 mm and 25.5% within 0.33–5 mm range, Figure 4(B) proportions greater than 5 mm were discarded (Masura et al., 2015). The presence of high proportions of secondary finer particles alludes to the breaking down of large plastic fragments (Zhang et al., 2015) which endorses our previous statement of biotic and abiotic degradation to smaller fractions that may result in underestimation of microplastics. These finer proportions together with other morphological characteristics (color and shape) may delude aquatic organisms and result in ingestion (Acosta-Coley and Olivero-Verbel, 2015; Cole et al., 2015; Veerasingam et al., 2016; Wang et al., 2017; Wagner and Lambert, 2018).

In our results, most abundant type of microplastic, 60%, was transparent, Figure 4(C) which can be attributed to the extensive use of transparent plastic for food and other packaging, and single-use shopping bags, remaining portion of microplastics consisted of colored particles with a majority of white 17.7%, blue 9.3%, and green 8.3%, which may be attributed to packaging, bottles, cosmetics, cleaning products, and clothing (Stolte et al., 2015).

Film was the most identified shape of microplastics according to results, Figure 4(A) which originate from weathering of larger plastic waste (Nor and Obbard, 2014). Fibers stood second on the list and probably have been the result of household sewage in the vicinity of tributaries, air deposition or disintegrated fishing nets (Browne et al., 2011; Cole et al., 2011; Wang et al., 2017). Granules were present in small numbers, which indicated cleaning products and cosmetics as the source (Cole et al., 2011), while foams in the samples might have resulted from packaging and building materials (PlasticsEurope & PEMRG, 2018; Rodrigues et al., 2018). Pictographic results are exhibited in Figure SD4.

### 3.4 Polymer categorization

Four types of polymers were identified in present analysis, Figure 4(D) Polyethylene (LDPE and HDPE) was identified as the predominant type of microplastic in Rawal lake and its tributaries which is coherent with previous studies. For example, PE and PP were the most abundant types of microplastic in Antuã River and Red Hills Lake (Rodrigues et al., 2018; Gopinath et al., 2020), PET and PE were dominant types found in Lake Ontario and urban surface waters of Wuhan (Ballent et al., 2016; Grbić et al., 2020; Wang et al., 2017), PE, PP, and PS were predominant in Japanese rivers (Kataoka et al., 2019) and nylon, PE, PS were leading microplastic type in Veeranam Lake (Srinivasalu et al., 2021). Around 78 particles of different shapes, colors, and sizes were carefully selected as representative of the visually identical fractions obtained from each sampling visit and subjected to FTIR analysis because of its extensive application in previous studies and reliability (Hidalgo-Ruz et al., 2012; Wang et al., 2017). Most of the selected particles were recognized as polyethylene followed by polypropylene and polystyrene, (Table SD1) which is consistent with previous literature and the fact that PE, PP and PS are largely produced single-use types of plastics (Bordós et al., 2019; Kataoka et al., 2019; PlasticsEurope & PEMRG, 2018; Rodrigues et al., 2018; Sruthy and Ramasamy, 2017; Zbyszewski et al., 2014) and have low specific gravity, which allows for their floatation on the water surface. The spectra of frequently identified particles are presented in Figure SD5. The dominance of polyethylene supports the fact that 55 billion plastic bags are produced per annum in Pakistan (WWF Pakistan, 2020). Polyester, synthetic fiber was also detected in lake surface water, which, however, was not detected in tributaries. So, it may be deduced that polyester and polystyrene were not originating from the tributaries as opposed to polyethylene and polypropylene, which were detected in the tributaries and were dominating in the lake as well, depicting that tributaries are contributing to microplastic concentrations in Rawal Lake. The incorporation of these microplastics into the lake may be lessened through appropriate measures in the environs of tributaries because they were found to originate from the tributaries. However, extensive survey and analysis should be carried in order to have the clarity of exact point sources of microplastics in lake in addition to the sources stated in this study and by (Tahira et al., 2020), and microplastics studies should be extended towards the Dam and the treatment plant at the outlet of lake to estimate about the discharging amount of microplastics from lake.

## 4 Conclusion

An estimation of microplastics in surface water of Rawal Lake and tributaries is presented in this study. The lake's surface water was found to be contaminated with microplastics, consistent with our assumption, but the concentration was lower than the previously conducted study at the lake, may be because of different methodologies and other environmental factors. The tributaries were found to be a contributing factor to the microplastics pollution in the lake by showing the presence of similar microplastic types as in lake. In tributaries, microplastics were present in downstream regions that are closer to the main city and have a high population. The primary sources were sewage waste and household dumping along the tributaries and main lake. The fine-sized microplastics were dominant in the lake, which, along with the abundance of secondary microplastics, implied the probable disintegration of larger plastic waste. The supreme traits of collected microplastics were films and transparent nature. Polyethylene (HDPE & LDPE) and polypropylene dominated the proportion of microplastics subjected to polymer identification.

Although, this study found relationship between microplastics in tributaries and in the lake. But this work is of exploratory nature given the limited sample size and temporal coverage. Therefore, it is recommended that future studies should be carried with maximum temporal and spatial coverage. To mitigate microplastics and anthropogenic particles entering the environment, decision-makers may begin by working to better understand and prevent these sources. For instance, educational programs that may control and prevent littering.

As also emphasized by literature, a standardized approach for sampling and processing is vital for comparison of results. In Pakistan, field of microplastics is in its nascent stage, and a standardized approach here may provide benefits for future studies and facilitate the comparisons of results locally. Future research in the field must also focus more on proposing viable solutions to the given problem, which is lacking in previous literature.

## Declarations

### Author contribution statement

Atif Bashir: Conceived and designed the experiments; Performed the experiments; Analyzed and interpreted the data; Contributed reagents, materials, analysis tools or data; Wrote the paper.

Imran Hashmi: Conceived and designed the experiments; Contributed reagents, materials, analysis tools or data.

### Funding statement

This work was supported by the 10.13039/501100007278National University of Sciences and Technology, Islamabad.

### Data availability statement

Data included in article/supplementary material/referenced in article.

### Declaration of interests statement

The authors declare no conflict of interest.

### Additional information

No additional information is available for this paper.
