# Supplementary material for: Detection in influx sources and estimation of microplastics abundance in surface waters of Rawal Lake, Pakistan
Source: Heliyon. 2022 Mar 24;8(3):e09166. doi: 10.1016/j.heliyon.2022.e09166 (PMC8965908; doi:10.1016/j.heliyon.2022.e09166)
Supplement: Supplementary data.pdf [file mmc1.pdf]

Supplementary Material

Detection in influx sources and estimation of microplastics abundance in surface waters of Rawal Lake, Pakistan

Atif Bashir*, Imran Hashmi

Institute of Environmental Sciences and Engineering, School of Civil and Environmental Engineering, National University of Sciences and Technology, Islamabad, 44000, Pakistan

***Corresponding author**

Atif Bashir

Institute of Environmental Sciences and Engineering, School of Civil and Environmental Engineering, National University of Sciences and Technology, Islamabad, 44000, Pakistan

Email: atifbashir52@gmail.com

Tel: +92-3046656554

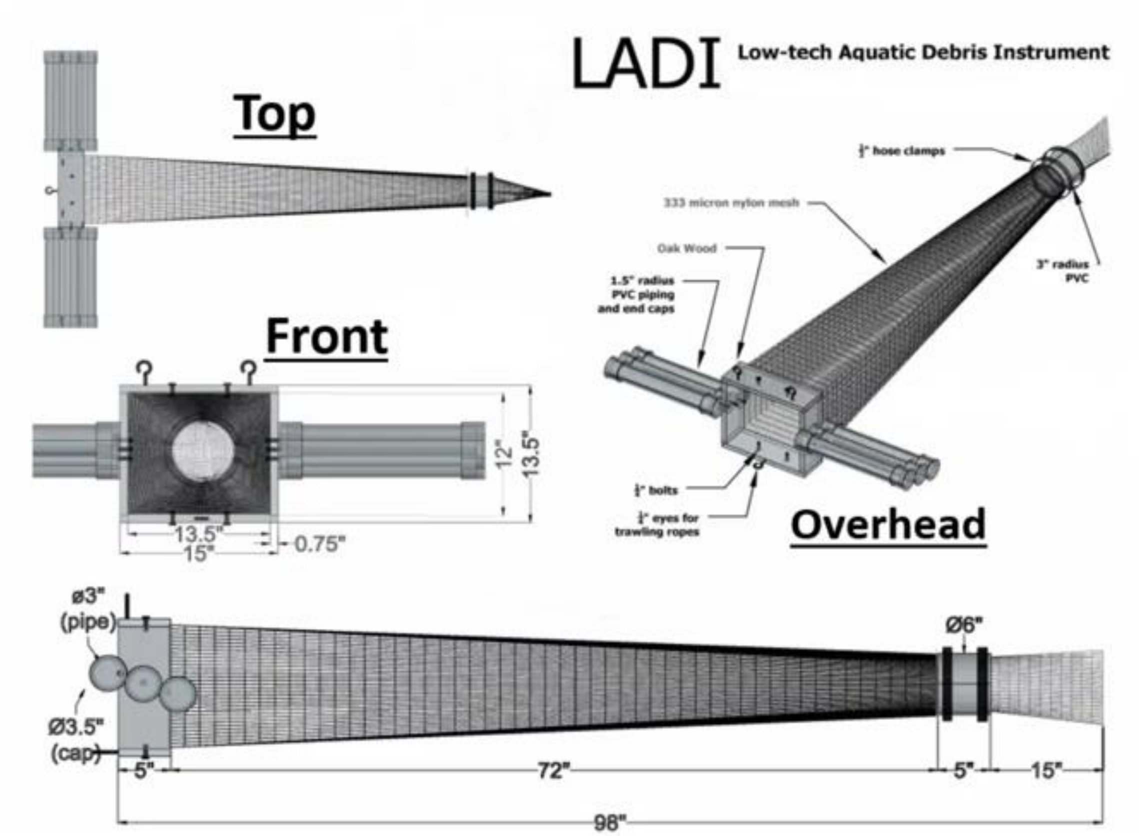

Figure SD 1. Build-it-yourself LADI (Low-tech Aquatic Debris Instrument) dimensions as proposed by (Coyle et al., 2016)

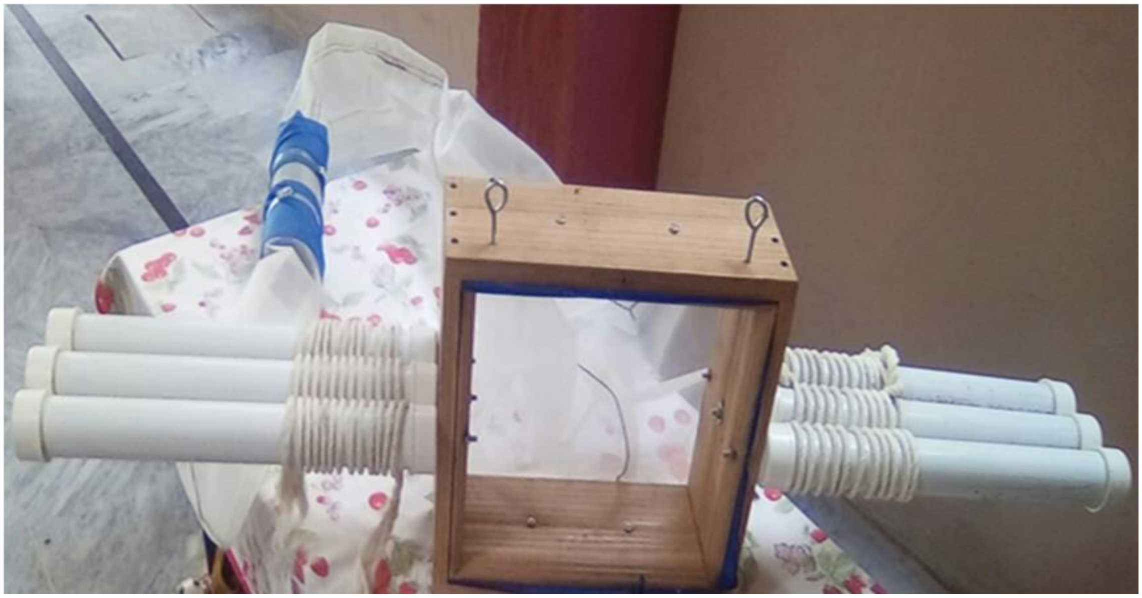

Figure SD2. Final designed trawl for Microplastic studies at IESE, SCEE, NUST, Pakistan.

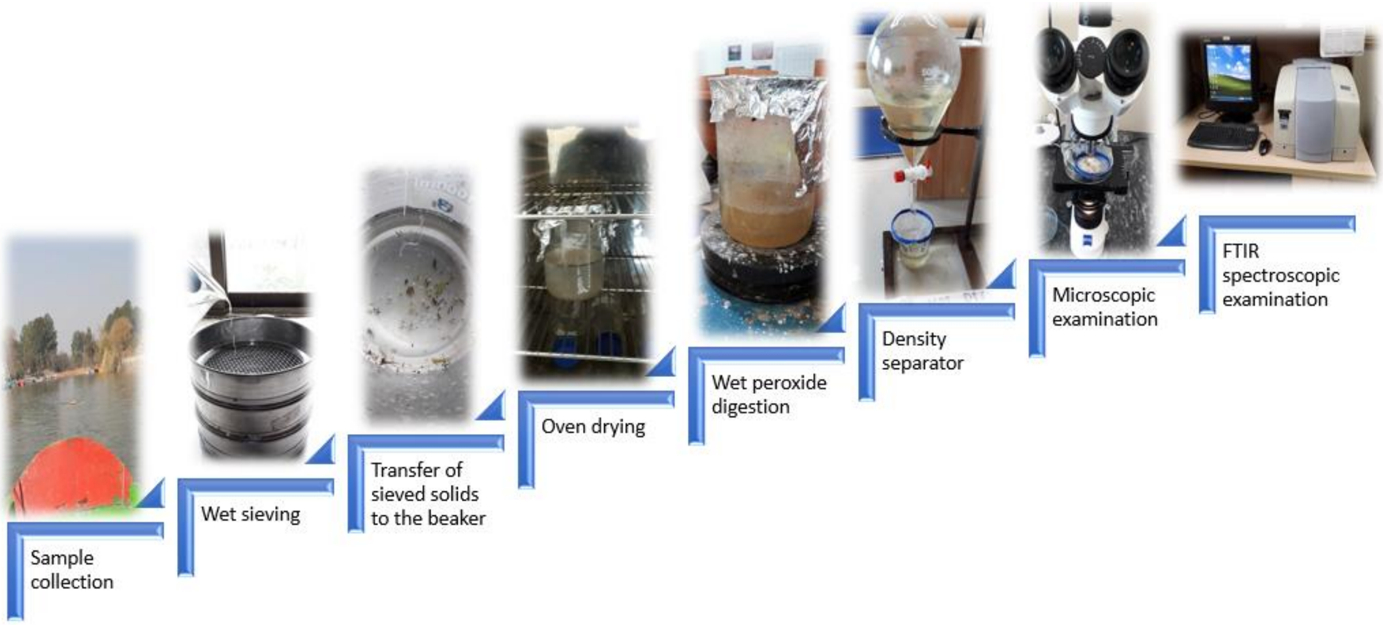

Figure SD3. Graphical representation of the methodology followed for the study for explicit understanding.

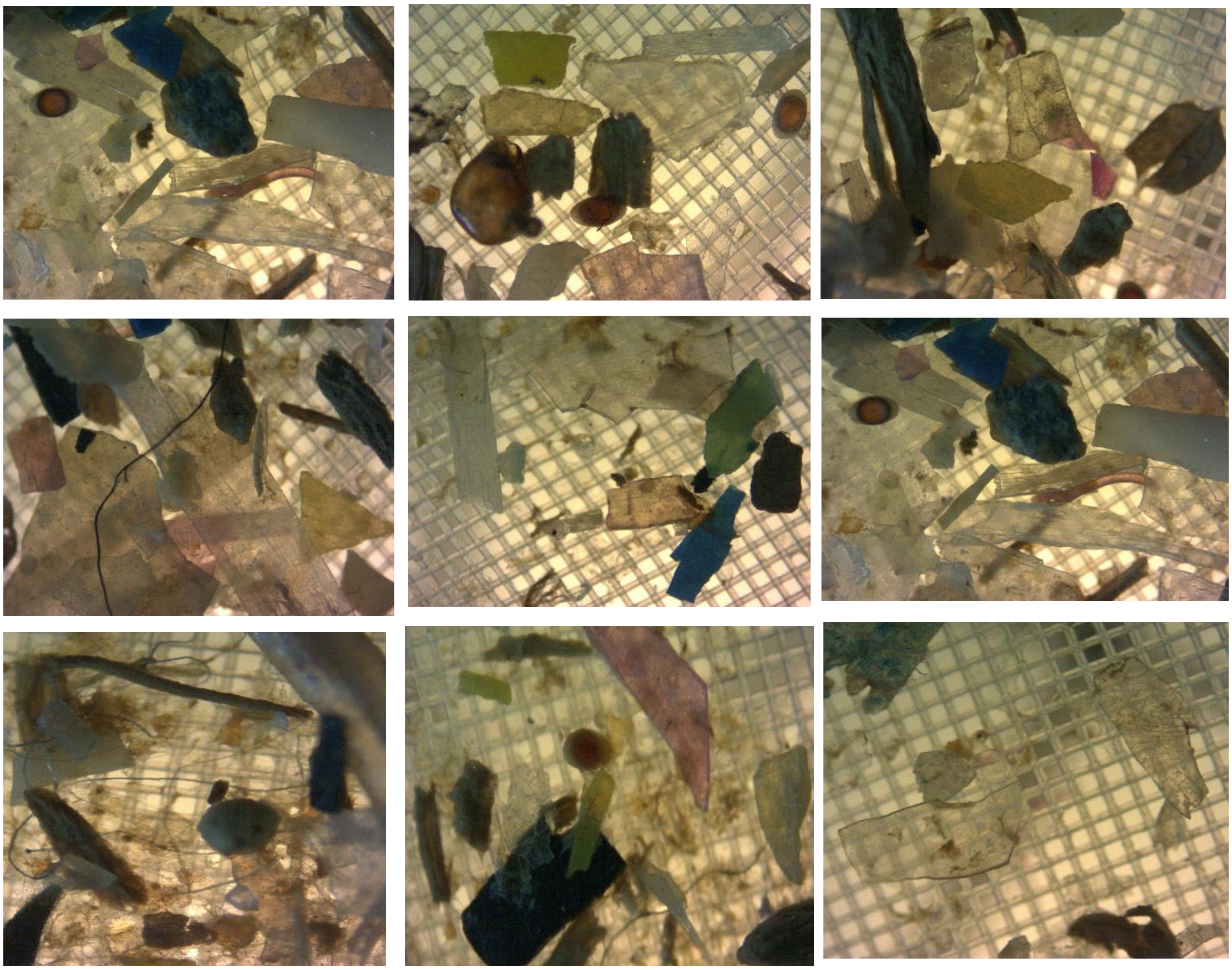

Figure SD4. Microscopic images of extracted microplastics.

Table SD1. Polymer type of selected microplastics for FTIR analysis.

	**Polyethylene**	**Polyester**	**Polystyrene**	**Polypropylene**	**Non-plastic**
Film	37		2	10	
Fiber	7	6		2	
Granule	4				1
Foam			9		
**Percentage**	61.5%	7%	14%	15%	1.3%

Figure SD5. FTIR spectra of identified microplastics.

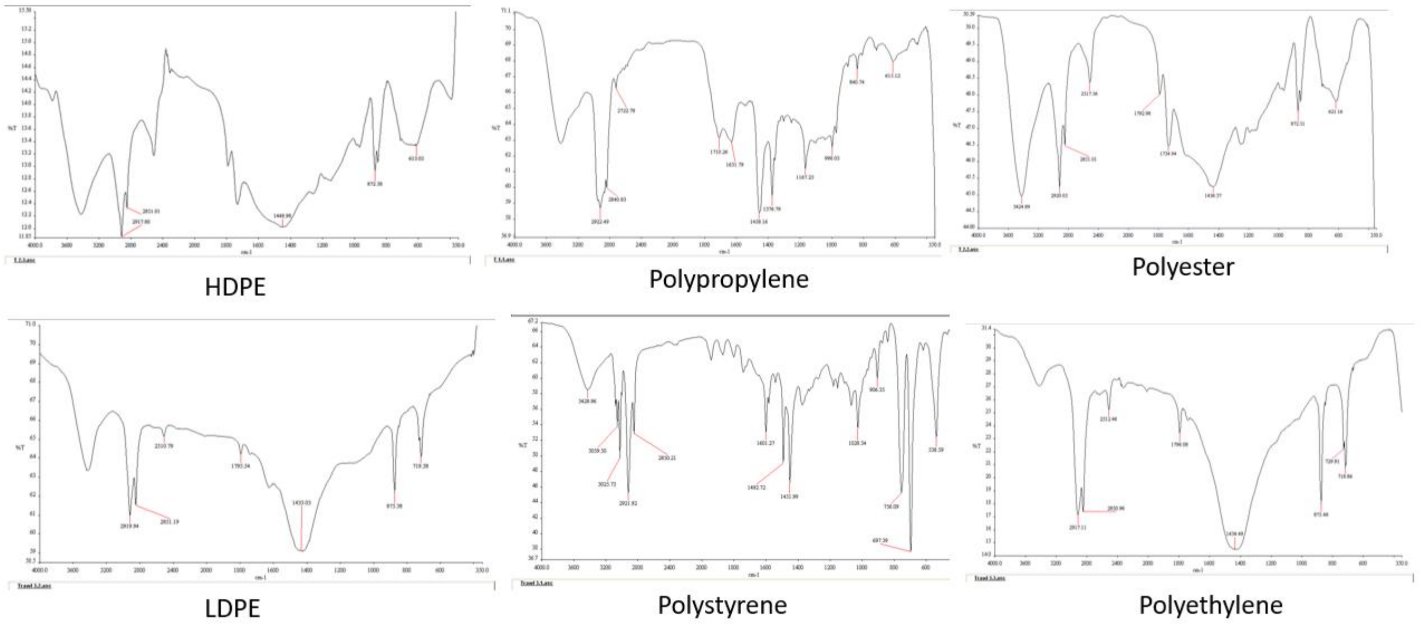

Table SD2. Microplastics concentrations in previously reported freshwater studies.

Study Area	Sampling method	Mesh size (μm)	Concentrations (particles/m³)	References
Lake Bolsena, Italy	Manta Trawl	333	0.82 to 4.42	(Fischer et al., 2016)
Urban surface waters of Wuhan, China	DC Teflon water pump	50	1660.0 ± 639.1 to 8925 ± 1591	(Wang et al., 2017)
Rhine River, Europe	Manta Trawl	300	1-20	(Mani et al., 2015)
Dongting Lake, China	DC Teflon water pump	50	900 to 2800	(Wang et al., 2018)
Lake Winnipeg, Canada	Manta Trawl	333	0.95-13.5	(Langen et al., 2017)
Hong Lake, China	DC Teflon water pump	50	1250 to 4650	(Wang et al., 2018)
Antuã River, Portugal	Motor water pump	55	58 to 1265	(Rodrigues et al., 2018)
Taihu Lake, China	Plankton Net	333	0.18-122	(Su et al., 2016)
Nakdong River, South Korea	Stainless steel beaker	20	293 to 4760	(Eo et al., 2019)
Carpathian basin, Europe	Jet pump	100	3.52 to 32.05	(Bordós et al., 2019)
Marne River, France	Plankton Net	80	5.7 to 398.0	(Dris et al., 2018)
Lake Chiusi, Italy	Manta Trawl	333	2.68 to 3.36	(Fischer et al., 2016)
Rawal Lake, Pakistan	LADI Trawl	100	6.4±0.5-8.8±0.5	Current study

Figure SD6. Pictorial outlook of the tributaries along with physical characteristics, **(A)** Ratahutar **(B)**Nurpur **(C)** Barakahu **(D)** Jinnah **(E)** Shadhara **(F)** Korang.

A.

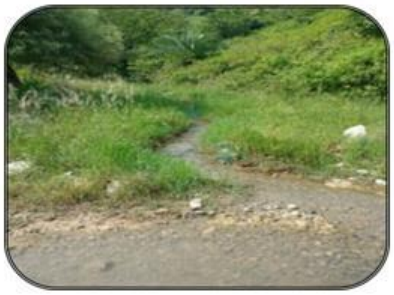

- No sewage discharges
- No waste dumps

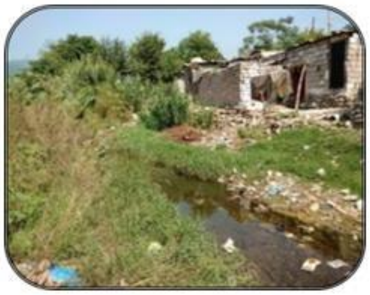

- Algal Growth
- Plastic Bags
- Waste Dump
- Animal Waste

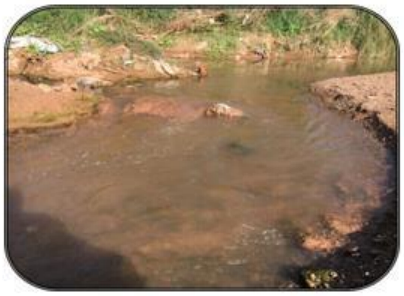

- Algal Growth
- Animal Waste
- Traces of household dumping

B.

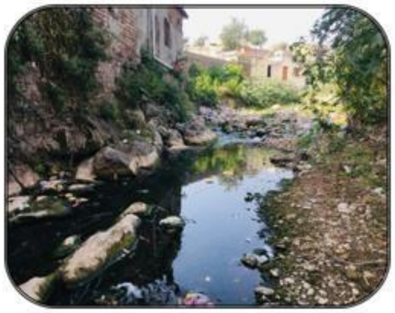

- Algal Growth
- Wastewater intrusion
- Plastic Bags
- Waste Dump
- Animal Waste

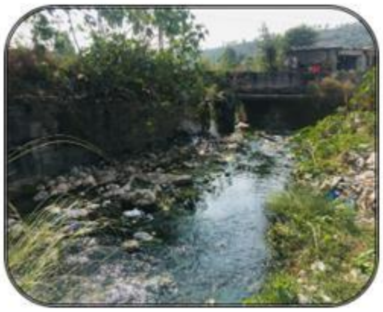

- Algal Growth
- Wastewater intrusion
- Plastic Bags
- Waste Dump
- Animal Waste
- Unpleasant odor

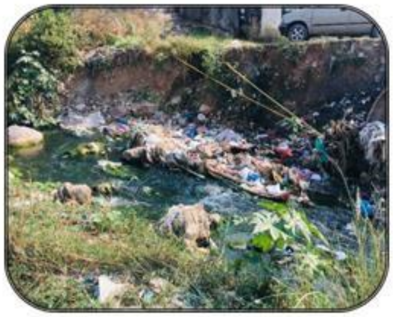

- Algal Growth
- Wastewater intrusion
- Densely populated area
- Plastic Bags
- Waste Dump
- Animal Waste
- Food waste

C.

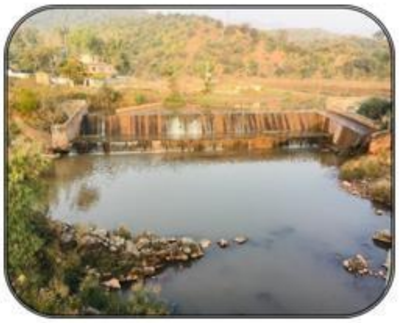

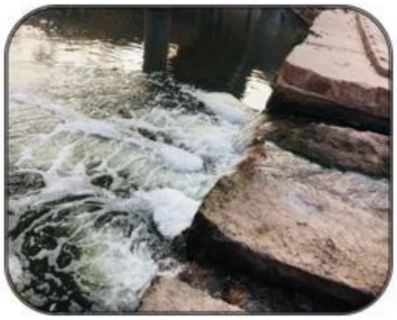

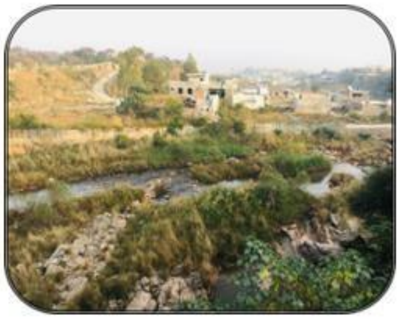

- Algal Growth
- Traces of waste dumping

- Wastewater intrusion
- Car washing
- Populated area
- Plastic Bags
- Animal Waste

- Algal Growth
- Wastewater intrusion
- Car washing
- Populated area
- Plastic Bags
- Waste Dump
- Animal Waste

D.

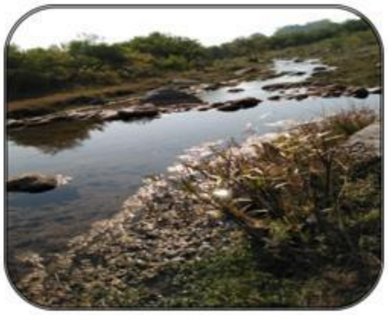

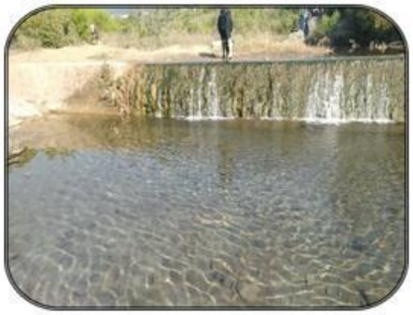

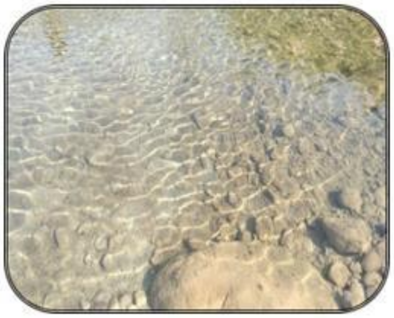

- Wastewater intrusion
- Heavy traffic nearby
- Construction waste
- Populated area
- Plastic Bags
- Animal Waste

- No sewage discharges
- Traces of algal growth

- No sewage discharges
- No waste dumps

E.

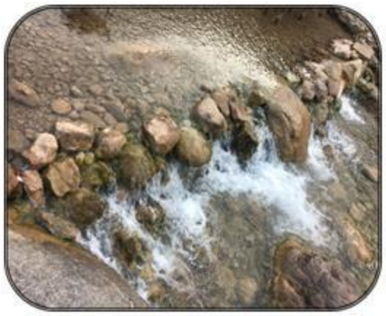

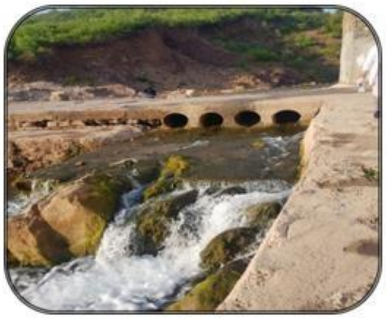

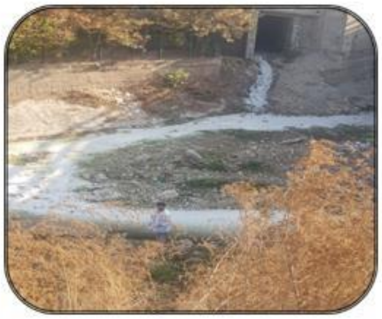

- No sewage discharges
- No waste dumps
- Clear water

- Algal Growth
- Traces of waste dumping
- Population settlements

- Wastewater intrusion
- Marble and kiln waste
- Poultry waste
- Heavy traffic nearby
- Construction waste
- Populated area
- Plastic Bags

F.

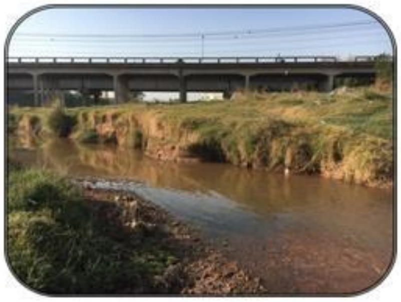

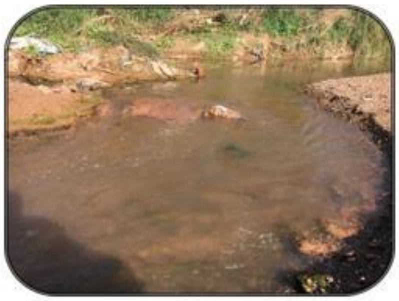

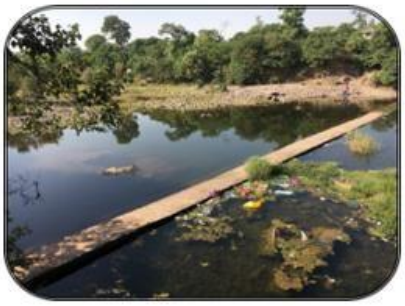

- Algal Growth
- Traces of waste dumping
- Wastewater intrusion

- Algal Growth
- Animal waste
- Muddy water

- Wastewater intrusion
- Household waste
- Heavy traffic nearby
- Populated area
- Plastic Bags
- Animal Waste
- Algal growth

Table SD3. Water quality indicators at the tributaries

Tributaries	Sampling Points	pH	DO (mg/L)	Temperature (°C)	BOD (mg/L)
Ratahutar Stream	Upstream	8.22	9.2	24.4	<1 mg/L
		8.49	8.6	24.6	
		8.54	8.5	24.7	
	Midstream	8.41	8.7	24.2	
		8.42	8.6	24.3	
		8.45	8.6	24.1	
	Downstream	8.40	8.30	24.4	
		8.42	8.40	24.3	
		8.37	8.40	24.0	
Nurpur Stream	Upstream	8.35	10.0	24.6	<1 mg/L
		8.23	9.54	24.2	
		8.11	9.39	24.3	
	Midstream	8.34	9.77	24.8	
		8.99	9.64	24.0	
		8.91	9.80	24.4	
	Downstream	8.22	8.1	24.1	
		8.73	8.31	24.5	
		8.81	7.93	24.3	
Jinnah Stream	Upstream	8.73	11.42	22.2	<1 mg/L
		8.40	11.00	22.4	
		8.64	11.32	22.1	
	Midstream	8.80	10.21	22.0	
		8.87	10.43	22.1	
		8.33	10.16	22.0	
	Downstream	8.95	9.1	22.4	>1 mg/L
		8.91	9.5	22.6	
		9.10	9.8	22.3	
Barakahu Stream	Upstream	8.90	8.55	21.9	<1 mg/L
		8.88	8.75	22.0	
		8.94	8.23	21.9	
	Midstream	8.99	7.43	22.3	
		9.10	7.80	22.7	
		9.15	7.27	22.6	
	Downstream	9.10	7.10	22.7	>1 mg/L
		9.45	7.16	22.8	
		9.23	6.92	22.5	
Shahdara Stream	Upstream	8.90	7.88	22.9	<1 mg/L
		8.82	8.1	23.2	
		8.91	7.90	23.0	

	Midstream	8.41	7.31	23.5	
		8.59	7.67	23.6	
		8.66	7.73	23.5	
	Downstream	8.39	7.1	23.5	>1 mg/L
		8.61	7.0	23.9	
		8.44	6.89	23.8	
Korang River	Upstream	7.90	6.9	24.0	<1 mg/L
		7.46	6.96	24.4	
		7.70	6.79	24.1	
	Midstream	8.0	5.89	24.5	
		8.12	6.10	24.3	
		8.23	6.12	24.6	
	Downstream	8.11	4.90	24.8	>1 mg/L
		8.30	5.43	24.8	
		8.16	5.1	25.0	

References

Bordós, Urbányi, B., Micsinai, A., Kriszt, B., Palotai, Z., Szabó, I., Hantosi, Z., & Szoboszlay, S. (2019). Identification of microplastics in fish ponds and natural freshwater environments of the Carpathian basin, Europe. *Chemosphere*, *216*, 110–116. https://doi.org/10.1016/j.chemosphere.2018.10.110

Coyle, C., Wells, E., & Liboiron, M. (2016). LADI and the Trawl. *Civic Laboratory for Environmental Action Research*, *August*, 80.

Dris, R., Gasperi, J., Rocher, V., & Tassin, B. (2018). Synthetic and non-synthetic anthropogenic fibers in a river under the impact of Paris Megacity: Sampling methodological aspects and flux estimations. *Science of The Total Environment*, *618*, 157–164. https://doi.org/https://doi.org/10.1016/j.scitotenv.2017.11.009

Eo, S., Hong, S. H., Song, Y. K., Han, G. M., & Shim, W. J. (2019). Spatiotemporal distribution and annual load of microplastics in the Nakdong River, South Korea. *Water Research*, *160*, 228–237. https://doi.org/https://doi.org/10.1016/j.watres.2019.05.053

Fischer, E. K., Paglialonga, L., Czech, E., & Tamminga, M. (2016). Microplastic pollution in lakes and lake shoreline sediments – A case study on Lake Bolsena and Lake Chiusi (central Italy). *Environmental Pollution*, *213*, 648–657. https://doi.org/https://doi.org/10.1016/j.envpol.2016.03.012

Langen, V. J., Challis, J. K., Anderson, P. J., Warrack, S., Langen, V., Challis, J. K., Hanson, M. L., & Rennie, M. D. (2017). Microplastic contamination in Lake Winnipeg , Canada Microplastic contamination in Lake Winnipeg , Canada *. *Environmental Pollution*, *225*(April), 223–231. https://doi.org/10.1016/j.envpol.2017.02.072

Mani, T., Hauk, A., Walter, U., & Burkhardt-Holm, P. (2015). Microplastics profile along the Rhine River. *Scientific Reports*, *5*(1), 17988. https://doi.org/10.1038/srep17988

Rodrigues, M. O., Abrantes, N., Gonçalves, F. J. M., Nogueira, H., Marques, J. C., & Gonçalves, A. M. M. (2018). Science of the Total Environment Spatial and temporal distribution of microplastics in water and sediments of a freshwater system (Antuã River , Portugal). *Science of the Total Environment*, *633*, 1549–1559. https://doi.org/10.1016/j.scitotenv.2018.03.233

Su, L., Xue, Y., Li, L., Yang, D., Kolandhasamy, P., Li, D., & Shi, H. (2016). Microplastics in Taihu Lake, China. *Environmental Pollution*, *216*, 711–719. https://doi.org/10.1016/j.envpol.2016.06.036

Wang, W., Yuan, W., Chen, Y., & Wang, J. (2018). Microplastics in surface waters of Dongting Lake and Hong Lake, China. *Science of The Total Environment*, *633*, 539–545. https://doi.org/https://doi.org/10.1016/j.scitotenv.2018.03.211

Wang, Wairimu, A., Li, Z., & Wang, J. (2017). Microplastics pollution in inland freshwaters of China : A case study in urban surface waters of Wuhan , China Science of the Total Environment Microplastics pollution in inland freshwaters of China : A case study in urban surface waters of Wuhan , China. *Science of the Total Environment, The*, *575*(September), 1369–1374. https://doi.org/10.1016/j.scitotenv.2016.09.213
